# Effect of different surface treatments on resin-matrix CAD/CAM ceramics bonding to dentin: in vitro study

**DOI:** 10.1186/s12903-022-02674-5

**Published:** 2022-12-23

**Authors:** Hanan Fathy, Hamdi H. Hamama, Noha El-Wassefy, Salah H. Mahmoud

**Affiliations:** 1 Conservative Dentistry Department, Faculty of Dentistry, Mansoura University, Mansoura, 35516 Egypt; 2 Dental Biomaterials Science Department, Faculty of Dentistry, Mansoura University, Mansoura, Egypt

**Keywords:** CAD/CAM, Hybrid ceramics, Indirect restorations, Inlays, Micro-tensile bond strength, Nano-ceramics, Polymer-infiltrated ceramics, Resin-based composites, Resin-matrix ceramics

## Abstract

**Background:**

Evaluating the effect of different surface treatment methods on the micro-tensile bond strength (µTBS) of two different resin-matrix computer-aided design/computer-aided manufacturing (CAD/CAM) ceramics (RMCs).

**Methods:**

A standardized inlay preparations were performed on 100 intact maxillary premolars. According to the type of the restorative material, the teeth were randomly divided into two equally sized groups (n = 50): (polymer-infiltrated ceramic (Vita Enamic) and resin-based composites (Lava Ultimate)). The inlays were fabricated using CAD/CAM technology. In each group, the specimens were randomly assigned to five subgroups (n = 10) according to the surface treatment method: group 1 used was the control group (no surface treatment); group 2, was treated with air abrasion with 50 μm Al_2_O_3_ (A) and universal adhesive (UA); group 3, was treated with air abrasion with 50 μm Al_2_O_3_ (A) and silane coupling agent (S); group 4, was treated with hydrofluoric acid (HF) and universal adhesive (UA) and group 5, was treated with Hydrofluoric acid (HF) + silane coupling agent (S). The inlays were then cemented to their respective preparations using dual-cure self-adhesive resin cement (RelyX U200, 3 M ESPE) according to the manufacturer’s instructions. The µTBS test was conducted in all groups, and stereomicroscope and scanning electron microscope were used to inspect the failure mode. The data were statistically analyzed using a two-way analysis of variance (ANOVA) and Tukey’s post-hoc multiple comparison tests at a significance level of *p* < 0.05.

**Results:**

Surface treatments significantly increased the µTBS of the materials compared to the control group (*p* < 0.05). For CAD/CAM RBCs, the µTBS value highest in group 2 whereas, for PICN, the µTBS value was highest in group 3. Cohesive failure of CAD/CAM restorative material was the most predominant mode of failure in all treated groups, whereas adhesive failure at restoration-cement interface was the most predominant failure mode in the control group.

**Conclusion:**

Surface treatments increase the µTBS of resin-matrix CAD/CAM ceramics to tooth structure. Air abrasion followed by universal adhesive and hydrofluoric acid followed by silane application appears to be the best strategies for optimizing the bond strength of CAD**/**CAM RBCs and PICN respectively.

## Background

Since 1990s, indirect esthetic restorations have gained popularity, and their use has increased significantly. They were introduced to overcome some of the drawbacks associated with direct filling techniques particularly in massively decayed or fractured posterior teeth [1–3].

With the availability of computer-aided design/computer-aided manufacturing (CAD/CAM) technology, indirect restorations with superior marginal and internal fit can be fabricated in a single appointment [4–6]. Also, the application of homogeneous industrial ceramic or composite blocks has resulted in fewer material failures during fabrication and clinical application [4, 7]. These blocks have fewer pores and flaws than do hand-built materials [8].

Ceramics and indirect resin-based composites have been widely used for indirect restorations because of their tooth-colored properties. However, the properties of the two materials differ greatly. Despite the remarkable optical properties and natural tooth-like appearance of the ceramics, their potential disadvantages include abrasion of the opposing dentition because of their high hardness and risk of brittle fracture and chipping [9, 10]. In contrast, resin composites cause less wear to the opposing dentition, are less brittle, are easier to repair and are more fracture resistant [11–13]. Nonetheless, their color stability is inferior, and the material wears down more quickly than ceramics [14].

Recently, Resin-matrix CAD/CAM ceramics (RMCs) have been developed. These materials combine the positive advantages of both ceramics and polymers [4, 15]. They have been referred to as “nano-ceramics” or “hybrid ceramics” but these terms do not accurately reflect the material’s percise chemical composition; therefore they are supposed to be only commercial terms [4, 16]. Due to the presence of both ceramic and polymer phases, RMCs are less brittle than cermaics, have superior flexural strength, machinability, and edge stability [17]. RMCs are further classified according to their industrial polymerization mode and microstructure into: high-temperature polymerized resin-based composites (RBCs) with dispersed ceramic fillers and high-temperature/high-pressure polymer-infiltrated ceramic network (PICN) [4, 18, 19].

CAD/CAM RBCs have a predominant organic phase and consist of a highly cross-linked polymeric matrix reinforced by nano or nano-hybrid ceramic fillers [4, 18]. Lava Ultimate, a CAD/CAM RBC material, is composed of silica and zirconia nano-fillers in the form of dispersed or aggregated particles (80 wt%) and urethane dimethacrylate (UDMA) as the resin matrix (20 wt%) [18, 20].

PICN material has a predominant inorganic phase [18]. It consists of a porous feldspar ceramic network that has been infiltrated by a polymer; therefore, it consists of two continuous interpenetrating networks, one composed of ceramic material and the other of the polymer [21]. The presence of two interconnected phases within PICN material, typically restricts crack propagation because of interfacial crack deflection [4, 21]. Vita Enamic (VE) is the PICN material currently available commercially. This material constructed by the infiltration of a pre-sintered glass-ceramic network (86 wt%) conditioned by a coupling agent with triethylene glycol dimethacrylate (TEGDMA) (14 wt%) by capillary action [20–22].

Effective adhesive bonding is crucial for the long-term success of indirect restorations because it minimizes the microleakage, improves marginal adaptation and increases the fracture strength [23–25]. There are two interfaces involved in the adhesive bonding of indirect restorations: the first is the interface between the tooth structure and resin cement which has been extensively studied and documented [26–28]. The second interface is the interface between the fitting surface of the indirect restoration and resin cement. Although adhesive bonding to dental ceramics has been extensively studied, data on the bonding properties of RMCs are limited [29, 30].

The industrial fabrication of RMCs using high temperature (˃100 °C) and/or high pressure polymerization (˃150 MPa) has significantly enhanced their mechanical properties [12, 16, 18, 31]. However, the high degree of conversion achieved has reduced the number of accessible free carbon-carbon double bonds on the material’s surface, which hinders its ability to bond with resin cement [11]. Therefore, the fitting surface of the restoration must be treated to obtain a reliable bond with resin cement [11, 32].Various surface treatment methods have been proposed to improve the bonding between the restoration and resin cement via micromechanical retention (e.g., alumina air abrasion or acid etching) or chemical bonding (e.g., silane coupling agent (S) or universal adhesive (UA)/resin primer) [18, 23, 33].

A systematic review was performed to determine whether CAD/CAM RMCs are clinically efficient materials for indirect restorations [34]. The results, indicated that CAD/CAM resin-based composite can be considered a reliable material for partial coverage restorations with clinical performance comparable to that of glass ceramic restorations [34]. However, the optimal surface treatment method is still debatable and showed variation among the reviewed studies. Also, research works that have been conducted to evaluate the effect of various surface treatments on micro-tensile bond strength (µTBS) between both types of RMCs and resin cements have been scarce so far. Therefore, the aim of the current study was to evaluate the effects of various surface treatment methods on µTBS between both types of RMCs and resin cement. The null hypothesis was that different surface treatment methods would not affect µTBS of resin-matrix CAD/CAM ceramic materials.

## Methods

### Materials

In this study, two different resin-matrix CAD/CAM ceramics were used: CAD/CAM resin-based composites (Lava Ultimate, 3 M ESPE, St Paul, MN, USA) and polymer-infiltrated ceramics (Vita Enamic, VITA Zahnfabrik, Bad Säkingen, Germany). The materials used are fully described in (Table 1).

**Table 1:** Materials used in the study

Material	Specification	Manufacturer	Batch number	Chemical composition
Lava ultimate	CAD/CAM resin-based composite	3 M ESPE, St Paul, MN, USA	N763594	Silica nanomers (20 nm), zirconia nanomers (4–11 nm), nanocluster particles derived from the nanomers (0.6–10 nm), silane coupling agent, resin matrix (Bis-GMA, Bis-EMA, UDMA, and TEGDMA)
Vita enamic	Polymer-infiltrated ceramics	VITA Zahnfabrik, Bad Säkingen, Germany	62,432	Ceramic component: SiO_2_ (58–63), Al_2_O_3_ (20–23), Na_2_O (6–11), K_2_O (4–6), B_2_O_3_ (0.5–2), CaO(< 1) and TiO_2_(< 1) Polymer component: methacrylate polymer Ceramic to polymer ratio; 86–14% by weight
RelyX U200	Self-adhesive Dual cure Resin cement	3 M, GmbH, Germany	6,690,509	Base paste: methacrylate monomers containing phosphoric acid groups, methacrylate monomers, silanated fillers, initiator components, stabilizers and rheological additives. Catalyst paste: methacrylate monomers, alkaline (basic) fillers, silanated fillers, initiator components, stabilizers, pigments and rheological additives
Single bond universal	Universal adhesive	3 M ESPE, St Paul, MN, USA	6,743,118	10-MDP, dimethacrylate resins, HEMA, vitrebond copolymer, filler, ethanol, water, initiators and silane
Porcelain primer/bis-silane	Pre-hydrolyzed silane primer	Bisco, Schaumburg, IL, USA	2,000,004,381	Silane with methacrylate (1–10%), acetone (30–70%) and ethanol (30–70%).
Porcelain etchant	Hydrofluoric acid-etch	Bisco, Schaumburg, IL, USA	2,000,001,191	9.5% hydrofluoric acid gel
N-etch	Phosphoric acid-etch	Ivoclar vivadent AG, Schaan, Liechtenstein	Y39063	37% phosphoric acid gel

*Bis-GMA* Bisphenol A diglycidyl dimethacrylate; *Bis-EMA* Ethoxylated bisphenol A dimethacrylate; *UDMA* Urethane dimethacrylate; *TEGDMA* Triethylene glycol dimethacrylate; *10-MDP* 10-methacryloxydecyl dihydrogen phosphate;*HEMA* Hydroxyethyl methacrylate

### Specimens selection

Sound extracted maxillary premolars were collected and a hand scaler (Zeffiro, Lascod, Florence, Italy) was used to remove calculus and soft tissue deposits. The teeth were then cleaned with a fine pumice water slurry and a rubber cup. All collected teeth were stored in 1% Chloramine-T solution as a disinfectant for 3 days. Of these collected teeth, 100 sound teeth free from cracks were selected after examination with a binocular stereo microscope (SZ TP, Olympus, Tokyo, Japan) at 30× magnification. The crown dimensions of the selected teeth as measured with a digital caliper, were as follows: 9.0–9.4 mm bucco-lingual length, 7.0–7.4 mm mesio-distal width and 7.7–8.8 mm cervico-occlusal height. During the study, the teeth were stored in distilled water at 37 ± 1 °C; the water was changed every 5 days with the use of incubator (BTC, BioTech Company, Cairo, Egypt). To prevent the teeth from dehydration, they were removed from the water only during the test procedures [1, 35].

### Specimens preparation

**Fig. 1:**
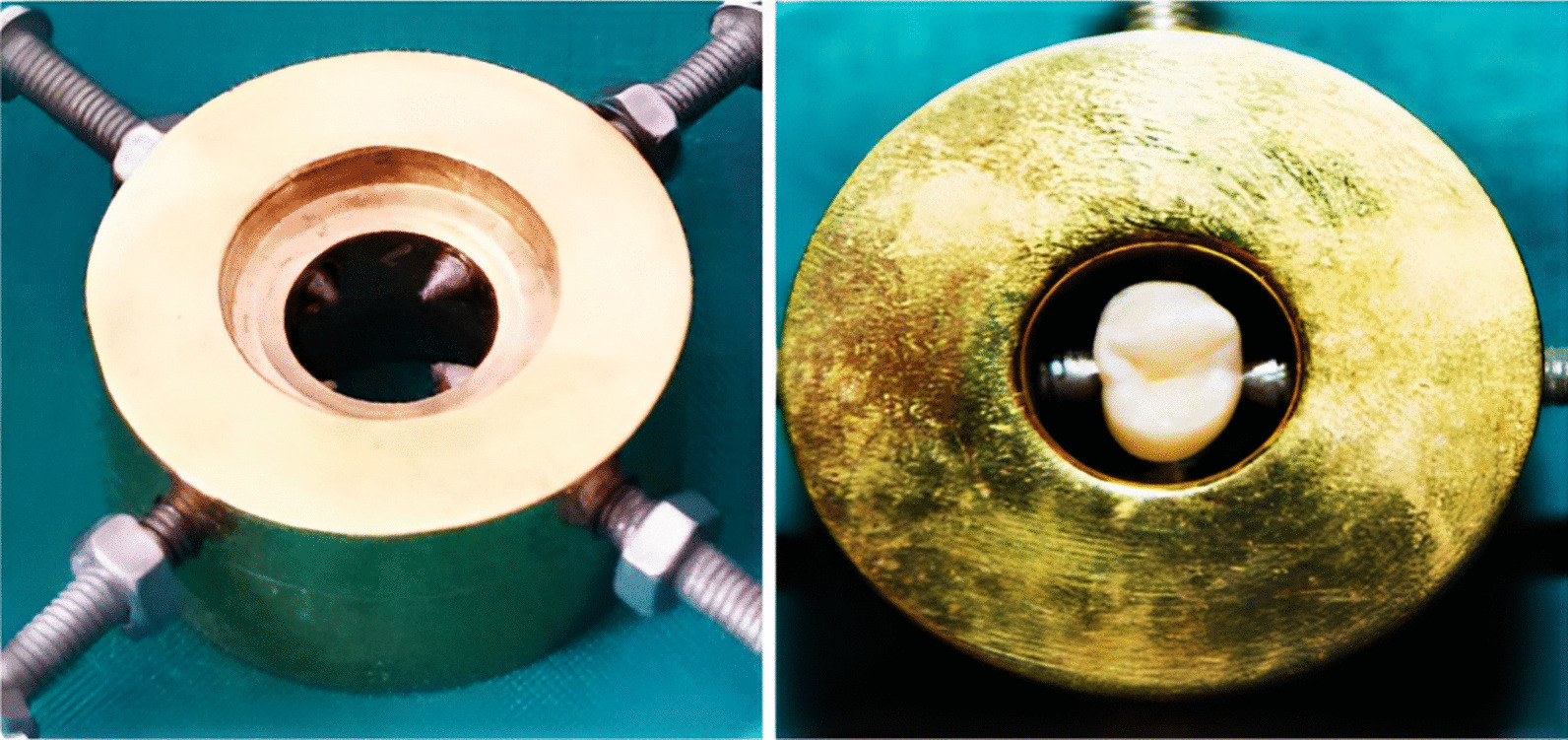
A specially designed device used for centralization of the tooth during fixation in PVC ring with auto-polymerizing acrylic resin

**Fig. 2:**
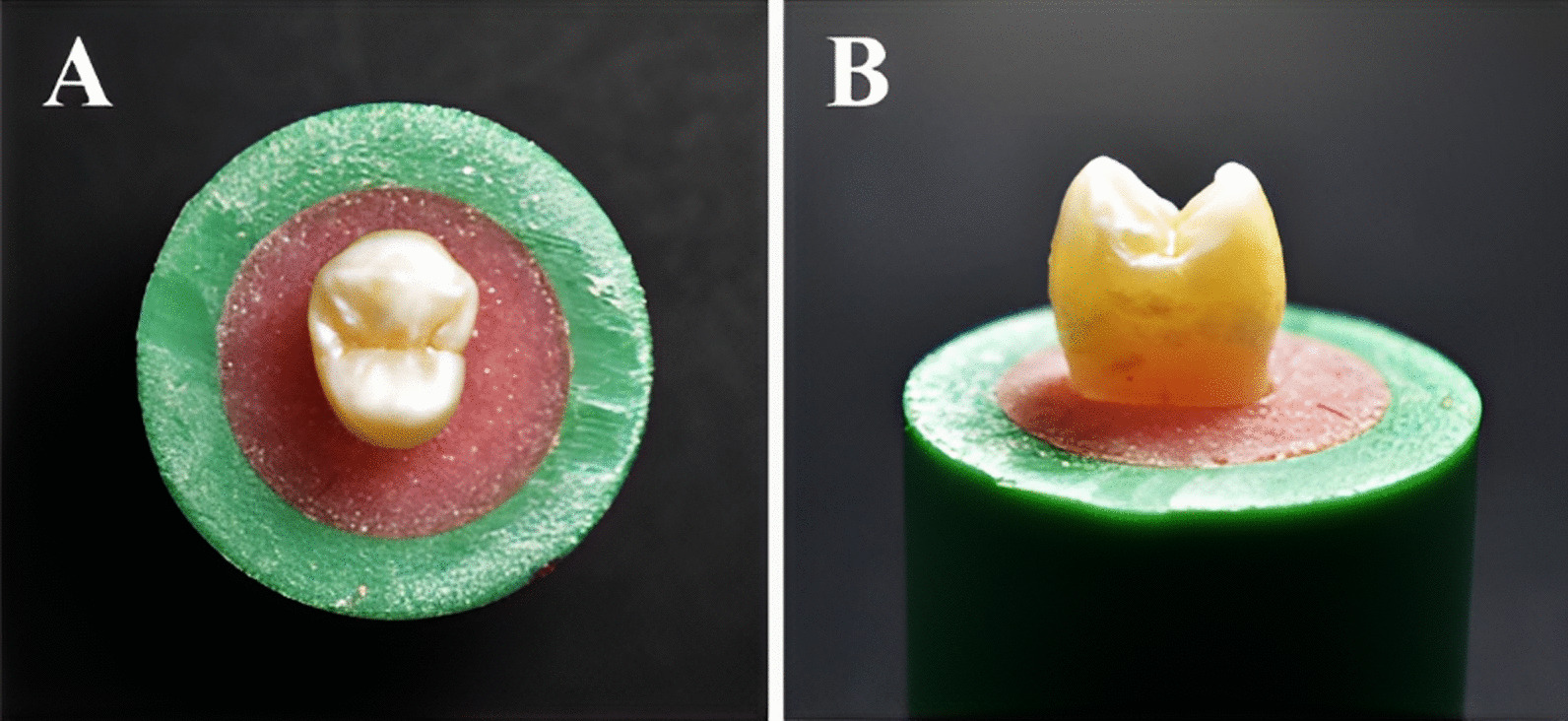
Selected tooth fixed in acrylic resin using PVC (Poly-vinyl chloride) ring **A** Occlusal view **B** Proximal view

The roots of the teeth were embedded in a cylindrical polyvinyl chloride (PVC) ring (1.4 × 2 cm) through the use of an auto-polymerizing acrylic resin (Acrostone, Cairo, Egypt) up to 2 mm below the cemento-enamel junction (CEJ). A centralization guide device designed at the Production of Engineering and Mechanical Design Department, Faculty of Engineering, Mansoura University was used to mount the teeth in acrylic resin cylinders (Figs. 1 and 2).

**Fig. 3:**
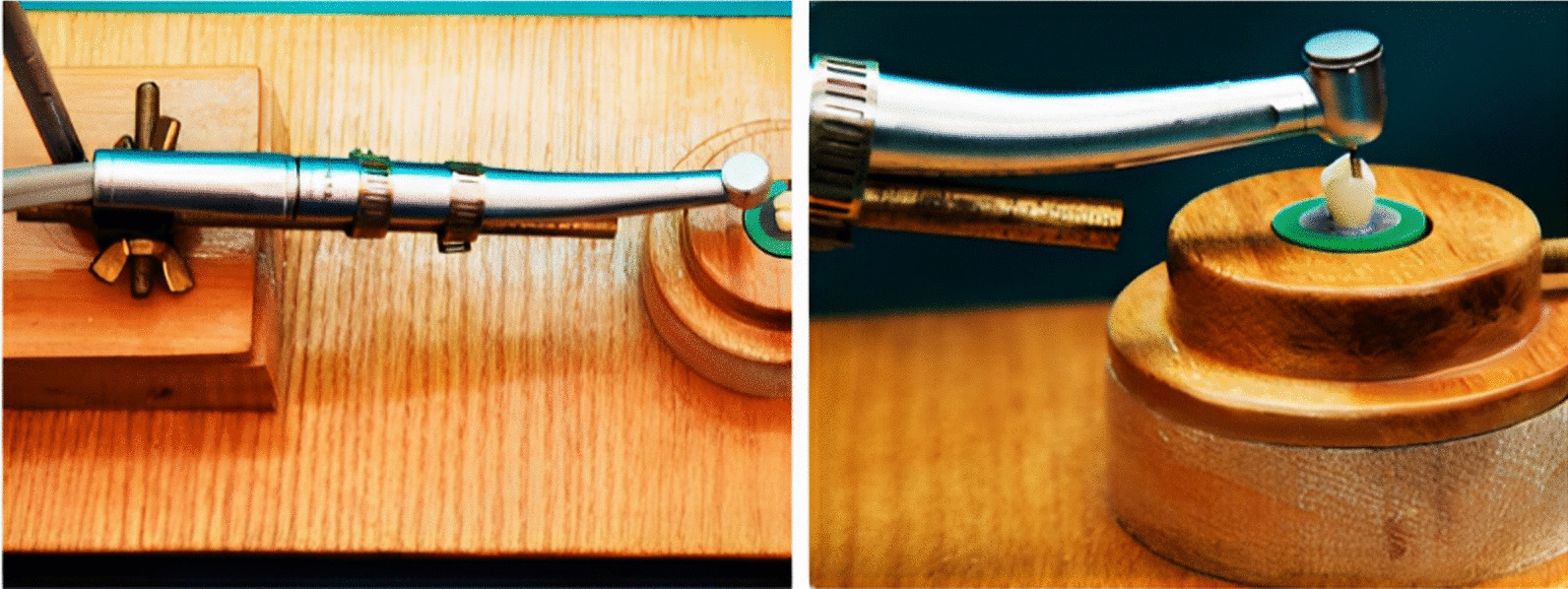
A specially designed apparatus to which the high-speed handpiece was affixed to standardize the cavity preparation

In a high-speed handpiece (W&H, Burmoos, Austria) under copious air-water cooling, a 6-degree tapered fissure diamond instrument (Inlay Prep Kit 4261, Komet, Lemgo, Germany) was used to create standardized inlay cavities. The same operator performed all the preparation steps according to the recommended sequence of specific diamond instruments. After five preparations, each used diamond instrument was replaced to ensure cutting efficacy. For standardized cavity preparation, the used handpiece was affixed to a specially constructed apparatus designed at the Production Engineering and Mechanical Design Department, Faculty of Engineering, Mansoura University (Fig. 3). This device enabled accurate handpiece movements, resulting in approximately standard divergence of the cavity walls with a standard depth and width [1].

**Fig. 4:**
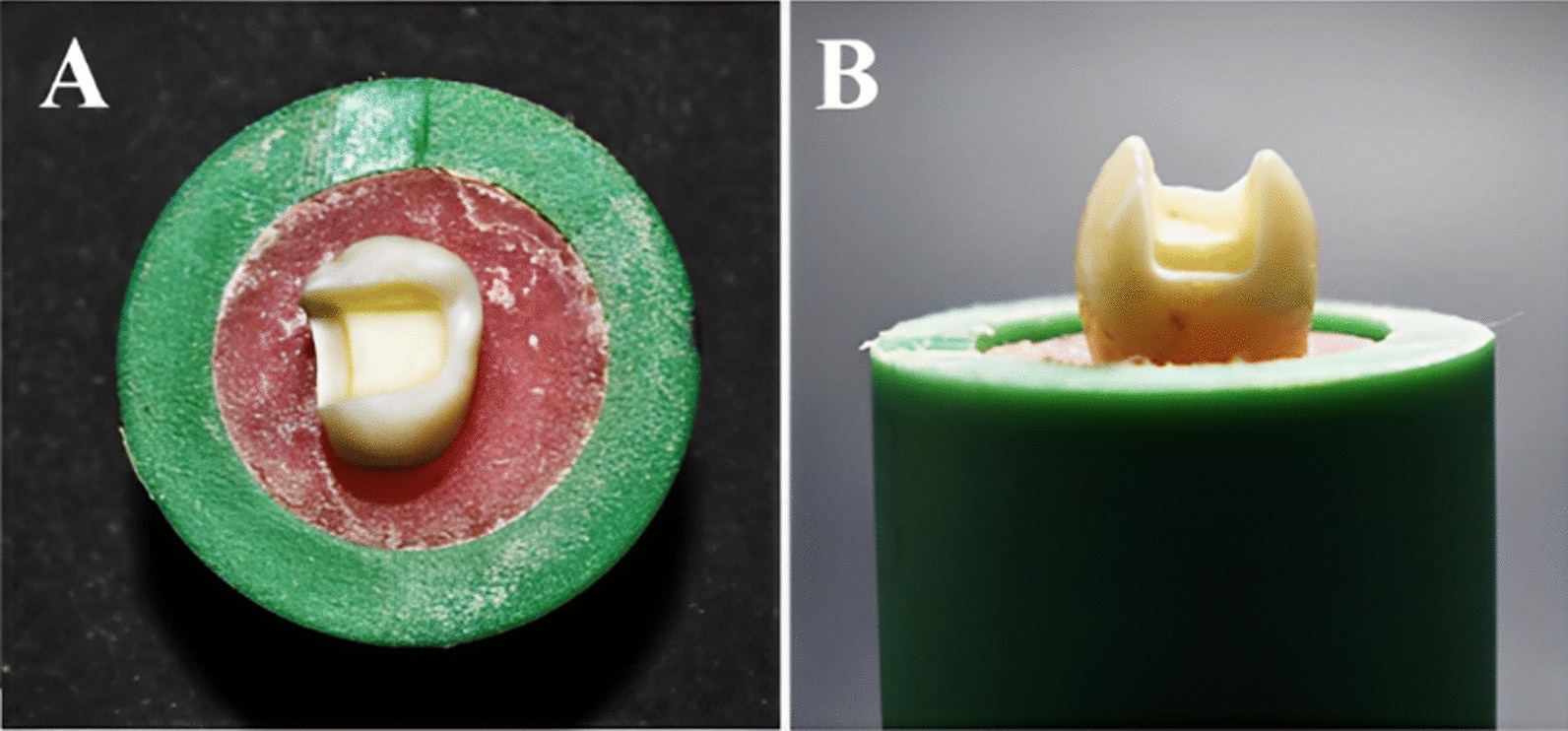
Tooth preparation **A** Occlusal view **B** Proximal view

The cavity preparation dimensions were as follows: the pulpal floor depth was 3 mm from the occlusal surface and the bucco-lingual width was more than half the inter-cuspal distance about 3.5 mm. Each proximal box had an axial wall height of 2 mm and a gingival floor depth of 1.5 mm (Fig. 4). The preparation had rounded internal line angles and butt joint cavo-surface margin. A digital caliper was used to measure the cavity dimensions. After each preparation, the prepared tooth was examined with 5× magnifying loupes (Univet, Rezzato, Italy) to exclude the existence of any disqualifying characteristics such as pulp exposures or cracks.

### Fabrication of the inlays

**Fig. 5:**
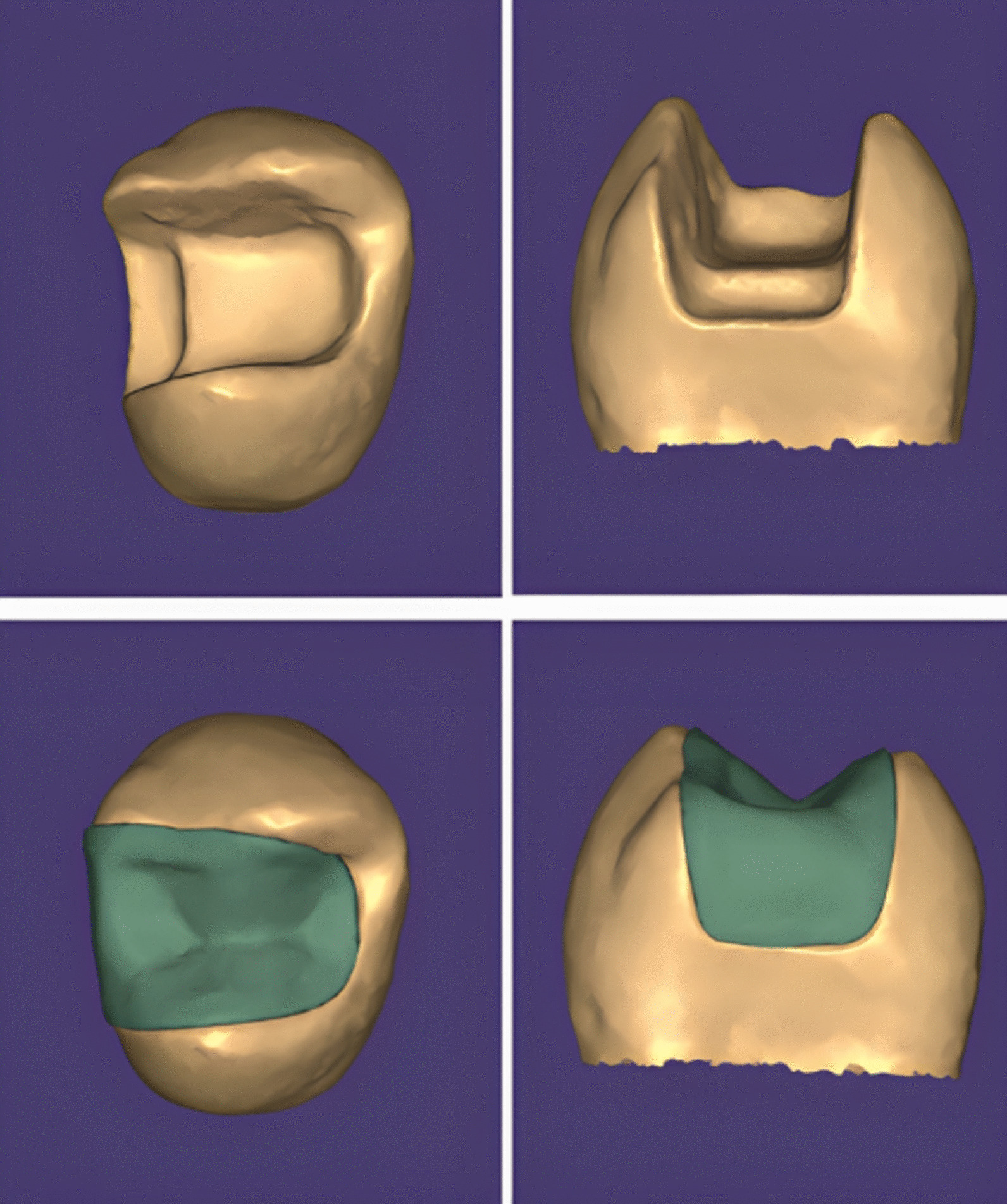
Scanning and designing process of inlay restoration using Exocad software (Exocad GmbH, Darmstadt, Germany)

Inlays were manufactured with CAD/CAM technology using Exocad software (Exocad GmbH, Darmstadt, Germany). Silver powder (CERCON, DeguDent GmbH, Hanau, Germany) was used on the prepared tooth surface to create an opaque surface needed for the scanning process using the CAD scanner (Ceramill Map 400, Amann Girrbach, Koblach, Austria). The anatomical configuration of the inlays was modified using CAD software (Fig. 5). The virtual die spacer was standardized (50 μm) across all groups. The milling process of the restorations was performed with (Ceramill motion 2, Amann Girrbach, Koblach, Austria).

Following the milling process, adequate fit of the restorations was confirmed using a vinyl polyether silicone material (Fit Checker-Advanced-Blue, GC Corporation, Tokyo, Japan) and restorations were cleaned in an ultrasonic cleaner with 99% isopropanol for 5 min. The prepared teeth were cleaned with pumice and rinsed thoroughly with water. The polishing procedure for PICN inlays was performed using the Enamic Polishing set (VITA, VITA Zahnfabrik, Bad Säckingen, Germany). RBC inlays were polished manually using diamond paste and silicon tips.

### Grouping of specimens

A two stage randomization was used for specimen’s allocation by using a table of random numbers. In step one, the specimens (n = 100) were randomly allocated into one of two main equal-sized groups (n = 50) depending on the type of the CAD/CAM restorative material utilized, those made of resin-based composites and those made of polymer-infiltrated ceramics. In step two, each group was further randomly divided into five subgroups (n = 10) based on the method of surface treatment used:

- Group 1: No surface treatment (No TTT): Negative control group.
- Group 2: Air abrasion and universal adhesive (A + UA): Air abrasion was performed with 50 μm Al_2_O_3_ at a distance of 10 mm at a pressure of 2 bar for 20 s. The specimens were then cleaned with alcohol, and dried with oil-free air. After air abrasion, Single Bond Universal Adhesive (3 M ESPE, St. Paul, MN, USA) was applied evenly to the cementation surface for 20 s and air-dried for 5 s according to the manufacturer’s instructions.
- Group 3: Air abrasion and silane coupling agent (A + S): After air abrasion as described for group 2, a silane coupling agent (Porcelain Primer/Bis-Silane; Bisco, Schaumburg, IL, USA) was applied to the cementation surface for 20 s and air-dried for 5 s according to the manufacturer’s instructions.
- Group 4: Hydrofluoric acid and universal adhesive (HF + UA): In accordance with the manufacturer’s instructions, 9.5% HF (Porcelain Etchant; Bisco, Schaumburg, IL, USA) was applied to the surface of each specimen for 60 s, followed by water rinsing for 60 s and drying using a water-free air spray. After acid etching, Single Bond Universal Adhesive (3 M ESPE, St. Paul, MN, USA) was applied evenly to the cementation surface for 20 s and air-dried for 5 s according to the manufacturer’s instructions.
- Group 5: Hydrofluoric acid and silane coupling agent (HF + S): After acid etching, as described for group 4, a silane coupling agent (Porcelain Primer/Bis-Silane; Bisco, Schaumburg, IL, USA) was applied to the cementation surface for 20 s and air-dried for 5 s according to the manufacturer’s instructions.

### Luting procedures

**Fig. 6:**
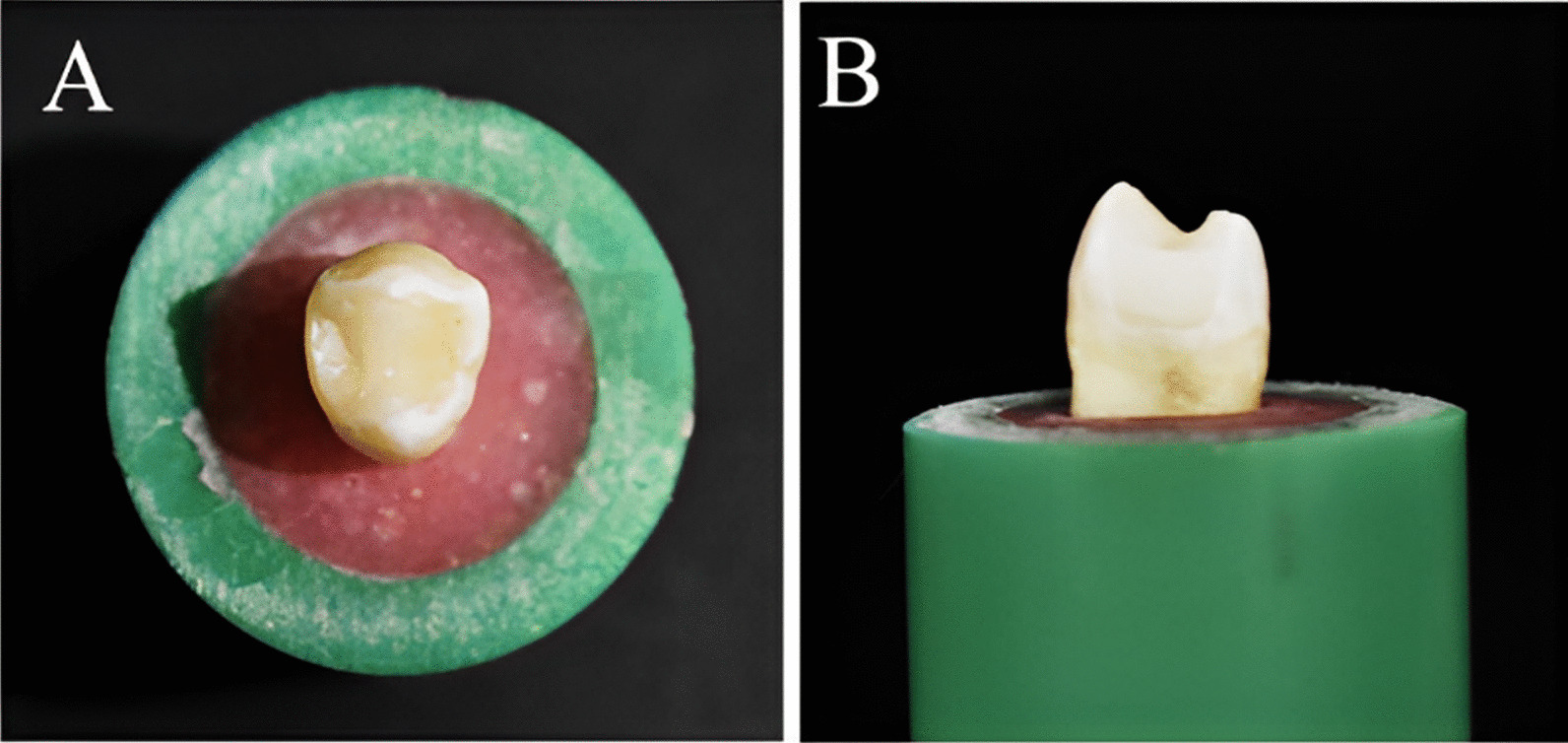
Final bonded specimen **A** Occlusal view **B** Proximal view

The enamel margins of all prepared teeth were selectively etched with 37% phosphoric acid (N-Etch, Ivoclar Vivadent AG, Schaan, Liechtenstein). To prevent the dentin from being etched, polytetraflouroethylene (PTFE) tape was applied to the dentin surface prior to the etching step. The inlays were then cemented to the tooth structure using self-adhesive dual-cure resin cement (RelyX U200, 3 M ESPE, St Paul, Minnesota, USA) according to the instructions of the manufacturer. The inlays were seated on the prepared teeth using finger pressure and then they were initially cured for 5 s at a distance of 2.0 mm using LED-curing unit (Elipar, 3 M ESPE, St Paul, Minnesota, USA) at an intensity of 1400 mW/cm^2^. After initial curing, a manual instrument was used to remove excess resin cement. Then an air-inhibiting gel (Glycerine) was applied along the margins to prevent the formation of un-polymerized resin layer. The restored teeth were then subjected to 30 s of light curing through the glycerine from each aspect. An example of the final bonded specimen after adhesive cementation is shown in (Fig. 6).

### Micro-tensile bond strength test

**Fig. 7:**
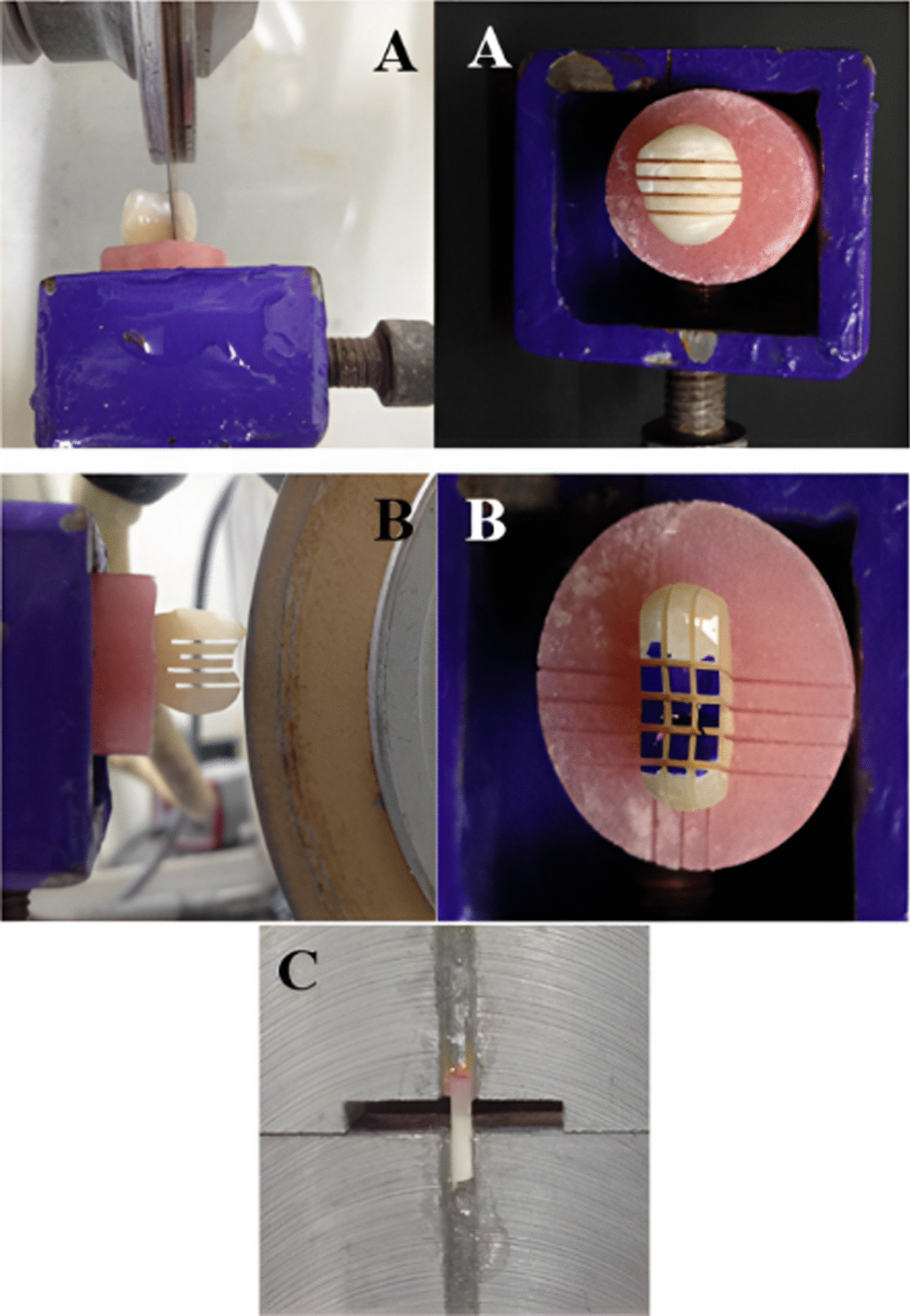
The specimen preparation for µTBS testing **A** The specimen was sectioned in occluso-gingival direction **B** The specimen was rotated 90° and cut was made perpendicular to occlusal surface to gain 1 ± 0.1 mm^2^ rectangular beams **C** Beam glued to Geraldeli’s jig

The restored teeth in the acrylic resin blocks were mounted in a diamond automated saw (Isomet 4000, Buehler Ltd., Lake Bluff, IL, USA). Using the low speed cutting saw, each specimen was sectioned in occluso-gingival direction under water cooling to produce 1-mm-thick slabs. In order to ensure that the slabs remained in place, the cutting was continued until 1 mm remained. The specimen was then rotated 90° and cut again perpendicular to the occlusal surface to gain 1 ± 0.1 mm^2^ rectangular beams. Each beam consisted of dentin and CAD/CAM restorative material with resin cement at the interface (Fig. 7). To obtain the beams, a final horizontal cut was made at the level of the cemento-enamel junction. Each beam was stored at room temperature in a plastic tight-seal cone containing distilled water and labelled with the tooth of origin and the subgroup.

Each beam was glued by its end in the central groove of Geraldeli’s jig using cyanoacrylate based glue (Zapit, Dental Ventures of America, Inc., Corona, CA, USA) (Fig. 7). Zapit accelerator was used to accelerate the hardening of the glue. Geraldeli’s jig was then mounted on a Universal testing machine (Instron, Norwood, MA, USA) and a tensile load was applied at a cross-head speed of 0.5 mm/min until the bond through the specimen failed. Bluehill Lite software (Instron, Norwood, MA, USA) then determined the µTBS in megapascals. Afterwards, specimen fragments were removed carefully from Geraldeli’s jig using a scalpel and stored in their respective labelled plastic cones until the failure mode was examined.

All specimens were inspected immediately after fracturing under a stereomicroscope (Olympus model SZ-PT, Tokyo, Japan) at 15× magnification to determine the failure mode. The fractures were categorized as: Type 1 (A-RC): adhesive failure at the restoration–cement interface, Type 2 (A-CT): adhesive failure at the cement-tooth interface, Type 3: cohesive failure in the restoration (C-R), Type 4: cohesive failure in the cement (C-C), Type 5: cohesive failure in the tooth structure (C-T), Type 6: mixed adhesive and cohesive in the restoration and cement (M-RC), Type 7: mixed adhesive and cohesive in the cement and tooth (M-CT). Representative fractured beams from each group were rinsed with ethanol and air-dried. The samples were then mounted on metallic stubs, gold sputtered (SPI Module—Sputter Carbon/Gold Coater, EDEN instruments, Japan) and inspected with scanning electron microscope (SEM) (JSM-6510 LV; JEOL Ltd., Tokyo, Japan) at 200× magnification.

### Statistical analysis

Sample size was calculated by Power Analysis and Sample Size Software (version 15, 2017), (PASS, NCSS, LLC. Kaysville, Utah, USA). A total of 100 intact maxillary premolars are required to provide 10 teeth per cell. This design achieves 98% power when an F test is used to test factor A (restorative material) at a 5% significance level and the effect size is 0.400, achieves 90% power when an F test is used to test factor B (surface treatment method) at a 5% significance level and the effect size is 0.400, and achieves 90% power when an F test is used to test the A*B interaction at a 5% significance level and the effect size is 0.400.

Data were tabulated and coded using Microsoft Excel 2016 (Microsoft Corporation, Redmond, WA, USA). Data analysis was performed using Statistical package for social science (SPSS 22, SPSS Inc, Chicago, IL, USA). The distribution of data was statistically checked with the Kolmogorov–Smirnov, and Shapiro-Wilk tests. A parametric two-way analysis of variance (ANOVA) was conducted followed by Tukey’s honestly significant difference (HSD) post-hoc multiple comparison test.

## Results

The Kolmogorov–Smirnov and Shapiro-Wilk tests revealed that all µTBS data followed a normal distribution pattern (*p* ˃ 0.05); therefore, a parametric two-way ANOVA was performed, which revealed that the bond strength was significantly affected by the type of restorative material and surface treatment method (*p* < 0.05). The interaction between these two variables was also statistically significant (*p* < 0.05).

Tukey’s HSD post-hoc multiple comparison test revealed that the surface treatments increased the µTBS of both CAD/CAM RBCs and PICN significantly compared to the control group (No surface treatment; *p* < 0.05). The µTBS mean values and standard deviations for all groups along with the results of Tukey’s HSD post-hoc multiple comparisons between groups are listed in (Table 2).

**Table 2:** Means (MPa) and Standard deviation (SD) values for different groups and multiple comparisons between means

	RBCs (lava ultimate)	PICN (vita enamic)
	µTBS (Mean ± SD)	µTBS (Mean ± SD)
No TTT (control)	6.39 ± 1.77^E^	5.44 ± 3.28^E^
A + UA	30.54 ± 4.29^ A^	16.22 ± 1.34^D^
A + S	20.63 ± 3.02^BC^	17.42 ± 2.14^CD^
HF + UA	23.56 ± 2.61^B^	20.93 ± 3.18^BC^
HF + S	17.46 ± 2.76^CD^	27.86 ± 2.69^ A^

Means are arranged from A to E with A are the highest values and E are the lowest values

Means with the same superscripted letters have no significant differences (Tukey HSD; *p* ˃ 0.05)

*RBCs* Resin-based composites; *PICN* Polymer-infiltrated ceramic network; *A* Air abrasion; *HF* Hydrofluoric acid; *S* Silane coupling agent; *UA* Universal adhesive

For CAD/CAM RBCs material, the µTBS mean value was highest in A + UA group, which differed significantly from the µTBS mean values of all the other test groups (*p* < 0.05). HF + UA group had the second highest µTBS mean value, which was significantly higher than HF + S group. In contrast, the difference was not statistically significant between HF + UA group and A + S group (*p* > 0.05). As well, there was no statistical significant difference between A + S group and HF + S group (*p* > 0.05).

For PICN material, HF + S group had the highest µTBS mean value, which was significantly higher than all other test groups (*p* < 0.05). HF + UA group had the second highest µTBS mean value, which was significantly higher than A + UA group (*p* < 0.05). Neither the values of HF + UA and A + S groups nor those of A + S and A + UA groups differed significantly (*p* > 0.05).

Tukey’s HSD post-hoc multiple comparison test also revealed a statistically significant difference in µTBS mean values between RBCs and PICN in A + UA and HF + S groups (*p* < 0.05). In A + UA group, CAD/CAM RBCs material recorded a significant higher µTBS mean value than PICN material, whereas, HF + S group, PICN recorded a higher µTBS mean value than did RBCs. The results also revealed no significant difference between the two materials in the control group, A + S group and HF + UA group (*p* > 0.05), although the CAD/CAM RBCs yielded more favorable results.

### Fracture pattern analysis

**Fig. 8:**
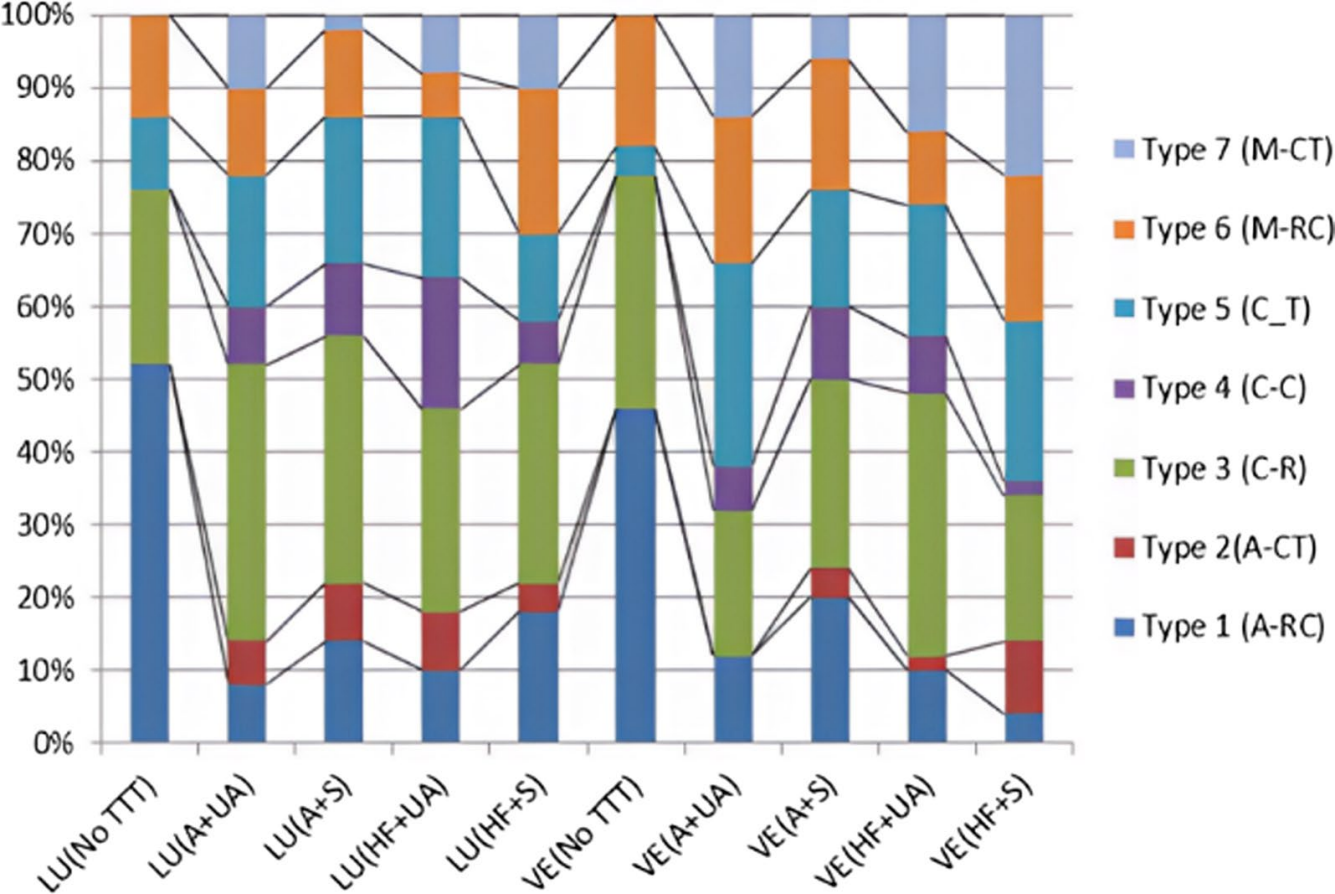
Failure patterns in percentages among tested groups

**Fig. 9:**
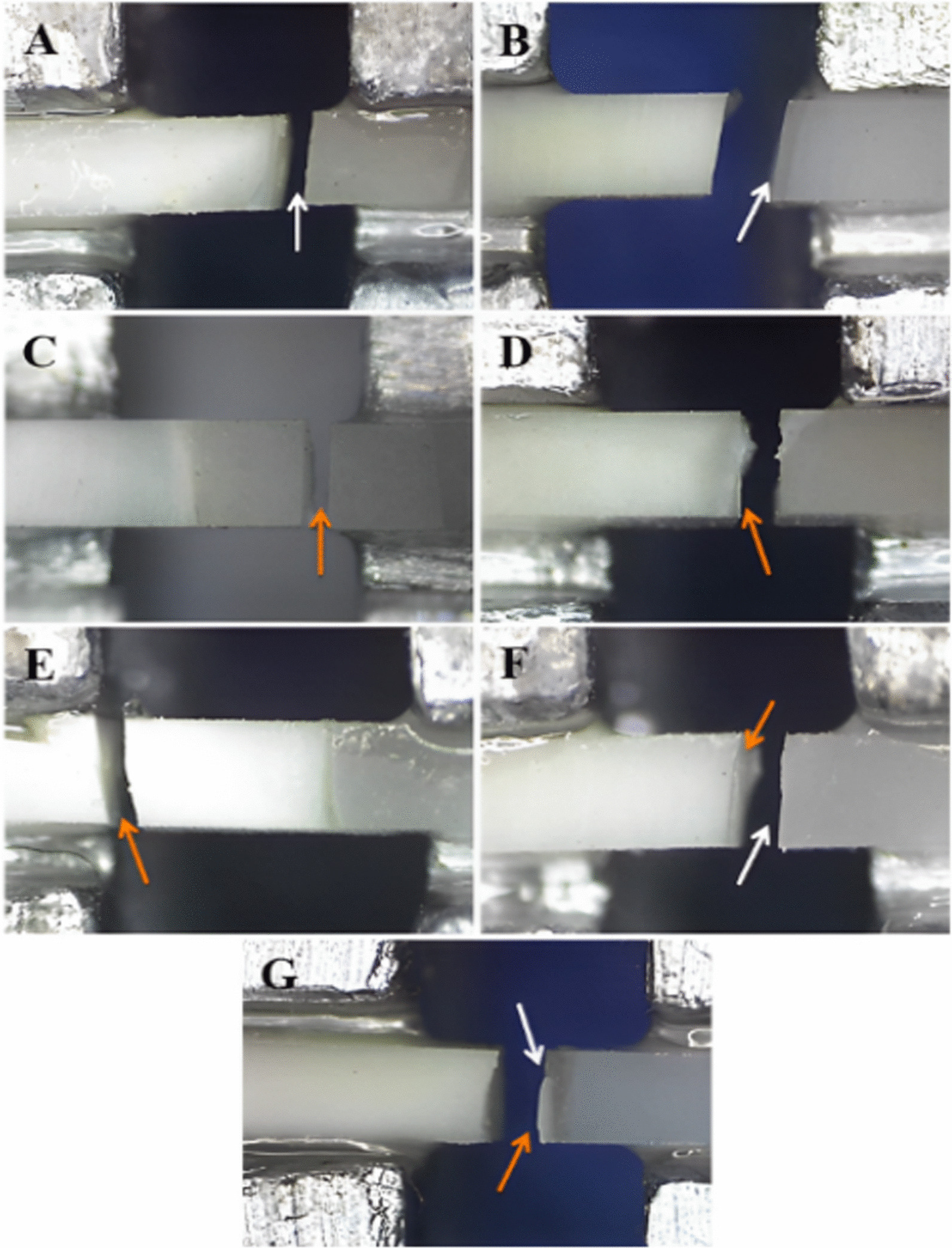
Stereomicroscopic views of fractured beams showing different failure patterns **A** Adhesive failure in the restoration-cement interface **B** Adhesive failure in the cement-tooth interface **C** Cohesive failure in the restoration **D** Cohesive failure in the cement **E** Cohesive failure in the dentin **F** Mixed adhesive and cohesive in the restoration and cement **G** Mixed adhesive and cohesive in the cement and dentin. White arrows represent adhesive failure; orange arrows represent cohesive failure

**Fig. 10:**
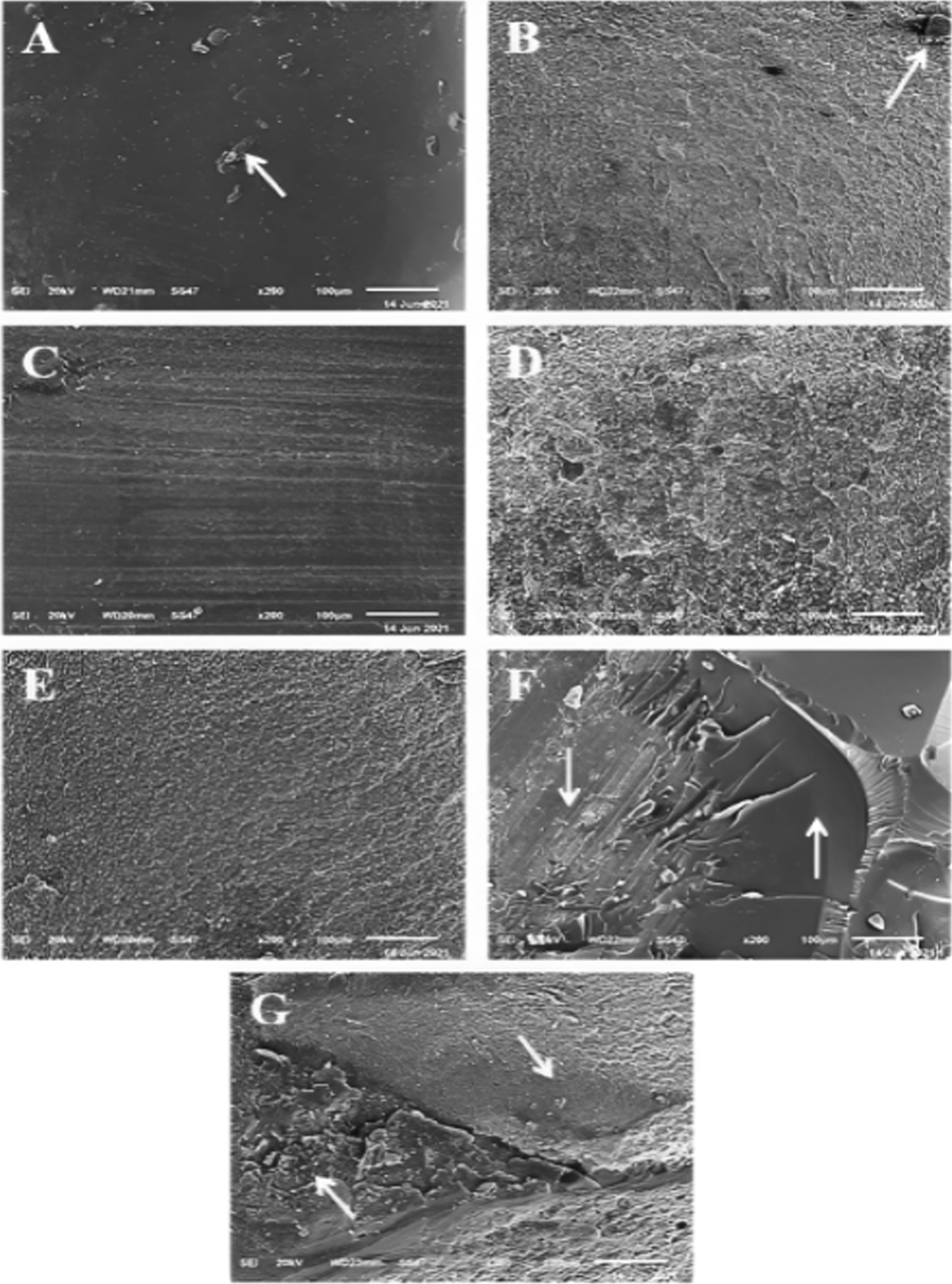
Scanning electron microscopic (SEM) micrographs of fractured beams showing different failure pattern. **A** Adhesive failure in the restoration-cement interface with some cement remaining on the restoration surface **B** Adhesive failure in the cement-tooth interface with some cement remaining on the tooth surface **C** Cohesive failure in the restoration **D** Cohesive failure in the cement **E** Cohesive failure in the dentin **F** Mixed adhesive and cohesive in the restoration and cement **G** Mixed adhesive and cohesive in the cement and dentin

In all tested groups, type 3 cohesive failure (fracture in CAD/CAM restorative material) was the most predominant mode of failure. Type 1 adhesive failure (at the restoration-cement interface) was most prevalent in the control group, whereas, cohesive and mixed fractures were most prevalent in all surface treated groups. The percentage values of failure pattern among all groups are illustrated in (Fig. 8), while different types of failure patterns are depicted in (Figs. 9 and 10) using stereomicroscopic and scanning electron microscopic images, respectively.

Ú

## Discussion

The results of this study, disproved the null hypothesis that different surface treatment methods would not affect the µTBS of resin-matrix CAD/CAM ceramic materials. The mean µTBS values of both materials increased significantly following the application of different surface treatments compared to when no surface treatment was applied.

The µTBS test was selected in this study because it allowed for a more precise evaluation of bond strength than conventional tensile and shear bond strength tests. Because stress distribution during shear testing is not homogenous, cohesive bulk fracture of the substrate away from the bonding interface occurs frequently [2, 36]. Therefore, the shear test offers limited insight into the true bond strength. The conventional shear and tensile bond strengths are also greatly affected by surface or internal flaws in the material. In the µTBS test, however, these defects are greatly reduced, allowing for a more homogenous and uniform stress distribution due to the small dimensions of the specimens and the small interfacial bonding zone [2, 36, 37]. Hence, the failure in µTBS occurs mostly at the adhesive interface enabling the recognition of the weakest part of the adhesive system [36, 38].

Since cohesive failure was the most common mode of failure among the experimental groups treated with A + UA, A + S, HF + UA and HF + S for both CAD/CAM materials, the results of the failure mode analysis supported the µTBS test results. In contrast, adhesive failure was the leading cause of failure among the untreated control group. This result is consistent with previous studies [23, 36]. Among mode of failures, cohesive failures exhibit the perfect bonding status that can be obtained as the failure arises mainly from flaws within the broken material and not at the interface, whereas, the main cause of adhesive failures is mostly low bond strength at the interface [36, 38–41].

In this study, various mechanical and chemical conditioning techniques were used. Mechanical conditioning via air abrasion or chemical etching using hydrofluoric acid have been shown to increase the surface energy and wettability of the restorative material by roughening their surfaces, thereby enhancing the mechanical interlocking between the CAD/CAM material and the resin cement [18, 42, 43]. Chemical conditioning with a silane coupling agent or universal adhesive (resin primer) has been shown to increase wettability, thereby facilitating the formation of covalent bonds between the restorative material and the resin cement [2, 23]. A recent systematic review and meta-analysis [18], found that the combination of mechanical and chemical surface treatment methods can enhance the positive effect of each protocol and increase the bonding strength of indirect restorative materials to resin cement.

This in vitro study demonstrated that surface treatments increase the µTBS between RMC restorations and tooth structure by enhancing the bonding to resin cement across all groups. This result is consistent with the results of previous studies [20, 23, 36, 44–49]. However, the optimal chemical and mechanical surface treatment methods for CAD/CAM RBCs and PICN materials differed significantly. This is likely due to the substantial difference in microstructure and composition between the two materials.

µTBS was greatest for PICN (Vita enamic) when the material treated with hydrofluoric acid followed by the application of a silane coupling agent. This result is consistent with manufacturer’s instructions and previous study findings [9, 36, 44, 47]. PICN material consisted of a dominant ceramic network infiltrated by a cross-linked polymer [22, 50]. Due to the microstructure and composition of the material, the ceramic content in the material was expected to guide the surface treatment.

Hydrofluoric acid tends to dissolve the glassy phase of the material, whereas the polymer network remaines intact [16, 18, 51]. The remaining polymer network creates a honeycomb structure and, therefore, a high micromechanical interlocking potential [18]. Silane coupling agent proved indispensable for use with silica-based ceramic materials because it enhanced the surface’s wettability and transforming it into hydrophobic surface, thereby enhancing the chemical bond to the resin cement [9, 52]. Consequently, the silanization process tends to condition the surface to bond to the resin matrix of the luting resin cement, resulting in an effective interaction [9, 44, 52]. Chemical conditioning PICN restorations with universal adhesive resulted in a lower µTBS results than chemical conditioning with a silane coupling agent. This is likely because the the universal adhesive contains insufficient silane to provide chemical adhesion to a silica-based ceramic surface [51, 53]. This hypothesis is supported by the findings of Abdou et al. [48], and confirmed in a study conducted by Rohr et al. [51], in which the application of universal adhesive in combination with a silane coupling agent yielded the highest bond strength in PICN material.

In this study, air abrasion groups failed to increase the µTBS of PICN material as effectively as HF. Nonetheless, the resulting bond strength was considerably greater than that of the control group. Subsequently, with the use of a scanning electron microscope, Motevasselian et al. [9] determined that after air abrasion, the surface of the specimen was relatively homogenous and not deeply pitted, whereas, in specimens that received HF, HF penetrated into the depth of PICN material by chemically reacting with the silica phase of the ceramic component of PICN. Also in the study by Elsaka et al. [36], the application of HF altered the surface texture of PICN material, resulting in the formation of numerous irregular and randomly distributed gaps and micropores, after HF application. Campos et al. [44],found that the bond strength was highest when CoJet Sand (3 M ESPE) and silane were used before aging. However, bond strength decreased significantly after aging, with HF and silane group achieving the highest values in this study.

For CAD/CAM RBCs (Lava ultimate), the combination of air abrasion and universal adhesive containing silane produced the highest µTBS in comparison with other surface treatment methods. This outcome was consistent with the manufacturer’s recommendations. In addition, previous studies demonstrated that alumina air abrasion provided greater bond strength than HF etching for CAD/CAM RBCs [11, 36, 45, 47, 54]. CAD/CAM RBCs are composed of a polymeric matrix reinforced by nano or nanohybrid ceramic fillers [18, 55]. The material, therefore, consists of two phases: a polymer matrix phase and an inorganic ceramic/glass phase [18, 56].

Hydrofluoric acid reacts with silicon dioxide and dissolves only the glassy phase of the CAD/CAM material, whereas alumina air abrasion increases the surface adherent area by roughening both the ceramic and polymer phases of the material [18]. Compared to PICN, CAD/CAM RBCs contains fewer silica particles that can chemically react with HF [18]. Elsaka et al. [36] discovered two continuous interpenetrating networks on the untreated PICN material’s surface topography: ceramic and polymer with micropores. The surfaces of the untreated CAD/CAM RBCs were more uniform and contained minute micropores. In the same study [36], the surface of CAD/CAM RBCs after air abrasion had well defined microscopically elevated and depressed areas, whereas, after HF treatment, the surface had only microscopic pores and pits. In an in vitro study, Peumans et al. [54] demonstrated that the after mechanical pretreatment with Al_2_O_3_ or CoJet Sand, bond strength was highest for Lava Ultimate and lowest for Vita Enamic.

µTBS results for CAD/CAM RBCs treated with universal adhesive were also superior to those treated with silane coupling agents. This finding was consistent with those in previous studies [11, 48]. The universal adhesive used in the current study consists of an acidic functional monomer [10-methacryloyloxydecyl dihydrogen phosphate (10-MDP)], methacrylate monomers and a silane coupling agent. The silane coupling agent provides chemical bond with silica-based ceramics [57] but it does not participate in any chemical bond with the polymer phase of CAD/CAM RBCs [18]. The 10-MDP monomer in the universal adhesive promotes bonding with zirconia, which is a component of the Lava Ultimate material [18, 58]. In addition, the acid groups of 10-MDP and the copolymer promoted bonding with the polymer phase of the material [18, 51]. In this manner, the universal adhesive tended to bond to both phases of the CAD/CAM RBC material and produced better results compared to silane coupling agents alone.

The current study had some limitations. For instance, only one type of adhesive resin cement was used (self-adhesive resin cement), necessitating the use of multistep luting agents in future studies. Additionally, during cementation, the use of finger pressure instead of applying standardized load. Furthermore, the absence of an aging procedure, such as storing the specimens in artificial saliva or thermocycling would allow for a more accurate simulation of real-life conditions. Bonding to the tooth structure may be another limitation of this study; failure often occurs at the tooth-cement interface rather than on the surface of the restorative material. The use of HF in various concentrations and air abrasion with different particle sizes should also be further investigated.

## Conclusion

Within the limitations of this in vitro study, the outcomes demonstrated that surface treatments increase the µTBS of RMCs, particularly RBCs, to tooth structures. The bond strength for PICN material was highest after the application of HF etching and a silane coupling agent. In contrast, the bond strength of CAD/CAM RBCs materials was highest after air abrasion, followed by the application of universal adhesive.

## Data Availability

The data sets used and/or analysed during the current study are available from the corresponding author upon reasonable request.
